# Influence of Novel Experimental Light-Cured Resin Cement on Microtensile Bond Strength

**DOI:** 10.3390/polym14194075

**Published:** 2022-09-28

**Authors:** Midori Kawamura, Yu Toida, Shuhei Hoshika, Md Refat Readul Islam, Yitong Li, Ye Yao, Yunqing Liu, Rafiqul Islam, Takaaki Sato, Yasushi Shimada, Hidehiko Sano

**Affiliations:** 1 Department of Restorative Dentistry, Graduate School of Dental Medicine, Hokkaido University, Kita 13 Nishi 7, Sapporo 060-8586, Japan; 2 Department of Dental Medical Laboratory, Hokkaido University Hospital, Kita 14 Nishi 5, Sapporo 060-8648, Japan; 3 Department of Restorative Dentistry, Faculty of Dental Medicine, Hokkaido University, Kita 13 Nishi 7, Sapporo 060-8586, Japan; 4 Department of Cariology and Operative Dentistry, Graduate School of Medical and Dental Sciences, Tokyo Medical and Dental University, 1-5-45 Yushima, Tokyo 113-8549, Japan

**Keywords:** bond strength, resin cements, filler morphology, mechanical properties, Knoop hardness, polymerization

## Abstract

The purpose of this study was to evaluate the microtensile bond strength (µTBS) and Knoop hardness number (KHN) of a novel experimental light-cured resin cement (HL). Eighteen flat dentin surfaces of human molars were polished using #600 SiC paper and bonded to CAD/CAM resin blocks with the respective resin cements and composites: HL, Panavia V5 (PV), and Clearfil AP-X (AP). All specimens were stored in distilled water at 37 °C for 24 h and 7 days. Scanning electron microscope (SEM) and energy dispersive X-ray (EDX) observations were performed to evaluate filler morphology and to detect the elements. The resin cements had a significant effect on the immediate µTBS (F = 22.59, *p* < 0.05) and after water storage µTBS (F = 22.83, *p* < 0.05). Significant differences (*p* < 0.05) in the KHN between the tested materials were observed, and HL indicated the highest KHN when compared with PV. HL showed a combination of the regular-shaped filler and spherical-shaped filler within the matrix. Silicon was detected in HL from the EDX evaluation. HL exhibited better bonding performance and polymerization, which may have contributed to the improvement of the adhesive strength.

## 1. Introduction

In the past three decades, various all-ceramic crowns have been developed, and indirect restorations, such as full-ceramic crowns, veneers, inlays, and onlays, have gained popularity [1] because of their outstanding esthetic characteristics, biocompatibility, durability, chemical stability, and high compressive strength in the oral environment [2]. Resin cement’s adhesion to tooth surfaces and restorative materials has been enhanced to improve fracture resistance and retention [3,4,5]. Resin cement can be categorized by bonding [6,7], including total-etch bonding, single-step etch bonding, self-cured resin cement, and dual-cured resin cement, and by polymerization, which includes self-cured, light-cured, and dual-cured polymerization [8,9]. Recently, dual-cured resin cement has become widespread for indirect restoration, providing the optimal combination of light-cured and chemical polymerization even amid inadequate irradiations [10,11].

In clinical situations, oral and sulcular fluids can cause cemented restoration failure due to resin cement’s water sorption, solubility, and microleakage. Furthermore, extrinsic and intrinsic discoloration can also affect esthetic restoration [12,13,14]. Water absorption and surface roughness are also responsible for extrinsic discoloration caused by food, drinks, and smoking [15,16,17], which can be managed by proper finishing and polishing [15]. Nevertheless, intrinsic discoloration is associated with the resin matrix and filler composition, the type of photo-initiator, polymerization systems, and the degree of conversion, which is not possible to manage clinically [18]. Most dual-cured resin cements contain unreacted benzoyl peroxide, which can cause discoloration and compromise the restoration’s esthetics [19]. Correspondingly, water sorption can degrade filler–matrix and induce swelling, thereby reducing a resin cement’s mechanical properties [20].

Recently, Kuraray Noritake Dental Corporation, Japan, developed a light-cured experimental resin cement, HL-100C (HL), with good color stability, sufficient working time, and newly developed spherical silica fillers for improved bonding stability. However, the novel experimental resin cement’s bonding performance and Knoop hardness have not been investigated to justify its clinical performance.

This study aims to evaluate the microtensile bond strength (µTBS) and Knoop hardness number (KHN) of an experimental light-cured resin cement with commercially available restorative resins. The null hypothesis of the study is that there is no significant difference between conventional and novel resin materials in terms of μTBS and KHN.

## 2. Materials and Methods

The Hokkaido University Research Ethics Committee approved the current project (approval number 2018-9). All teeth were placed in a 0.5% Chloramine-T solution at 4 °C and immersed in distilled water for 30 min before use.

### 2.1. Materials Used in the Study

A novel experimental light-cured resin cement (HL), a commercially available dual-cured resin cement, Panavia V5 (PV), and a resin composite, Clearfil AP-X (AP), were used in this study. Additionally, PV tooth primer (PV-Primer) and Clearfil Se Bond 2 (SE2) were used. All materials were obtained from Kuraray Noritake Dental, Tokyo, Japan. The materials used in this study and their composition are shown in Table 1.

### 2.2. Specimen Preparation

The specimens were prepared using eighteen caries-free human molars and CAD/CAM (12 sizes; shade A3LT) resin blocks (Katana Avencia Block 2, Kuraray Noritake Dental, Tokyo, Japan). The CAD/CAM resin blocks were sliced into 1.5 mm thickness using a slow-speed diamond saw, Isomet (Buehler, IL, USA). The CAD/CAM resin block surfaces were polished under running water for 60 s with 600-grit SiC sheets (Sankyo-Rikagaku Co., Ltd., Tokyo, Japan), sandblasted with 50 μm alumina powder at 0.2 MPa, ultrasonically cleaned for 2 min, and then dried with a syringe air blow for 10 s. All CAD/CAM resin block samples were etched with phosphoric acid and a silane coupling agent, following the manufacturer’s instructions.

The flat, occlusal dentin surfaces of the molar teeth were exposed using a model trimmer under water cooling and then polished with #600 SiC paper for 60 s under running water to produce smear layers for bonding. All samples were divided into three groups according to the resin materials: HL-100C (HL), Panavia V5 (PV), and Clearfil AP-X. (AP). For the HL and PV groups, PV-Primer was applied to the exposed surface, according to the manufacturer’s recommendations. For the AP group, the primer and bonding agent SE2 were applied to the exposed dentin surface. Then, the resin materials were applied to the tooth surface, and the prepared CAD/CAM resin slices were placed with firm pressure over the resin cement or resin composites. The samples were light-cured at ≥2000 mW/cm^2^ for 20 s using a cordless LED (Pencure2000, Morita, Tokyo, Japan).

**Figure 1:**
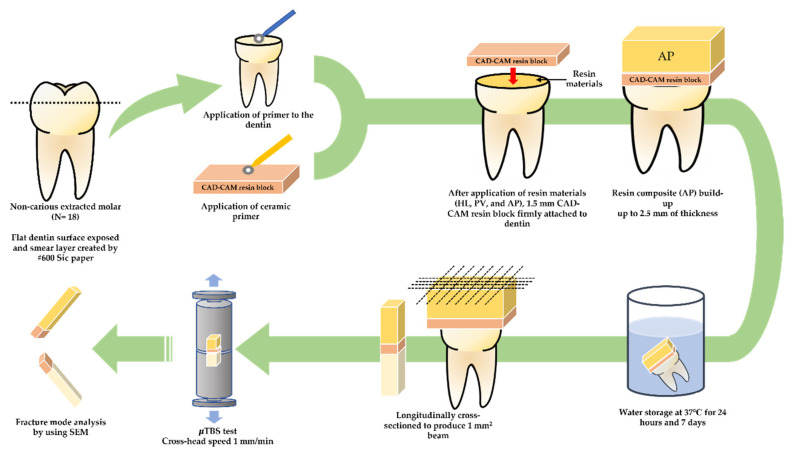
Graphical representation of specimen preparation and experimental set-ups for evaluating µTBS and fracture mode.

To obtain the standard height for the µTBS test, the CAD/CAM resin block surface was applied with Clearfil Ceramic Primer Plus (Kuraray Noritake Dental, Tokyo, Japan) for the HL and PV groups. The SE2 priming agent was applied for the AP group, followed by a bonding agent and light curing. All groups received 2.5 mm of a light-cured AP resin composite. The specimens were stored in 37 °C water for 24 h and 7 days. A graphical representation of the study design is presented in Figure 1. 

### 2.3. µTBS Test

After storage, the specimens were longitudinally cross-sectioned with an Isomet (slow-speed diamond saw) to produce a beam-shaped specimen with a surface area of around 1 mm × 1 mm. Before testing the µTBS, the digital caliper (E-PITA15, Nakamura Mfg. Co., Ltd., Chiba, Japan) was used to measure each beam size and record the results. After that, the specimen was fixed to the Ciucchi’s jig using a cyanoacrylate adhesive (Model Repair 2 glue, Dentsply-Sankin, Tokyo, Japan) and placed in a testing device (EZ-S test, Shimadzu, Tokyo, Japan). Then, tensile force was applied at a cross-section speed of 1 mm/min until it fractured. Fifteen beams were tested for each group.

### 2.4. Fracture Modes Analysis

Following the µTBS test, the fractured specimens were air-dried, mounted on an aluminum stub, sputter-coated with Pt–Pd for 120 s, and analyzed using an SEM (S-4000, Hitachi, Tokyo, Japan) at ×80 and ×1000 magnifications with an accelerating voltage of 5 kV. The failure modes were classified into six categories: type 1, interfacial failure at block (IB); type 2, interfacial failure at block and cement (IB + C); type 3, interfacial failure at block, cement, and dentin; type 4, cohesive failure in cement (CC); type 5, interfacial failure at dentin and cement (ID + C); type 6, inter facial failure at dentin (ID).

### 2.5. Filler Morphology Observation by Scanning Electron Microscope (SEM)

The specimen blocks for each resin were made with polypropylene tubes (2 mm in thickness and 13.8 mm in diameter). Then, a glass slide was placed on top and light-cured for 20 s. The filler morphologies were observed on polished and unpolished samples. For polished samples, the resin surfaces were thoroughly polished with SiC paper (#600, #1000, #1200, and #2000), followed by a diamond polishing paste with grit sizes of 6 μm, 3 μm, 1 μm, and 0.25 μm for 60 s each. The samples were then ultrasonically cleaned for 5 min. For unpolished samples, the resin material groups were submerged in acetone for 30 s. The samples were cleaned with water, air-dried for 10 s, and kept in a plastic container. The specimens were air-dried and sputtered with a Pt–Pd ion while mounted to aluminum stubs. At 5 or 10 kV, the specimens were evaluated with an SEM. The filler particle sizes were detected at ×2000, ×5000, ×20,000, and ×50,000 magnifications. Image J (National Institutes of Health, Bethesda, DC, USA) was used to examine filler particle morphology.

### 2.6. Atomic Elemental Analysis by Energy Dispersive X-ray (EDX)

The cured resin blocks of all groups (3 mm × 3 mm × 2 mm in size) were fabricated and embedded in an epoxy resin. After 24 h, the embedded blocks were polished with #600, 800, and 1000 SiC papers under running water. Afterward, the surfaces were polished using 6 µm, 3 µm, and 1 µm diamond pastes (DP-Paste, manufactured by Struers in Copenhagen, Denmark). An ultrasonic device was used to clean the surface. The elemental analysis of the cured resins was carried out using (EDX) mode equipped with an SEM (JSM-5310LV, JEOL, Tokyo, Japan) at ×3000 magnification with an acceleration voltage of 20 kV. This process was carried out after the resin cement sample had been allowed to dry in a plastic container for 24 h.

### 2.7. Knoop Microhardness Test

Five resin samples were prepared to measure the KHN. The samples were made employing a 13.8 mm length, 13.8 mm width, and 2 mm depth Teflon mold. The resin materials were dispensed directly into the Teflon mold. The excess resin was removed, and a 25 µm mylar strip was placed on top. The resin was then cured for 40 s using a PENCURE2000 at 2000 mW/cm^2^. During the light activation procedure, the light guide was placed in the middle of the specimen. All procedures were performed at (23 ± 2 °C) and (50 ± 10%) humidity. This prevented photo-initiator sensitization. In addition, a filter was utilized to maintain the ambient red light. The resin cement samples were kept in a dry, dark environment at 37 °C for 24 h after light curing.

Microhardness was tested using a microindenter (MVK-C, Akashi, Kanagawa, Japan) with a ×20 objective lens and 25 gf load for 15 s. Each specimen was indented ten times at 0.5 mm intervals, starting from the center and proceeding outward. Each indentation’s long-axis length was measured to calculate the KHN.

### 2.8. Statistical Analysis

The results of µTBS and KHN were analyzed using a one-way ANOVA, and a Games–Howell test was performed at a level of significance of 5%. IBM SPSS Statistics Version 22 for Windows (IBM, Tokyo, Japan) was used to perform the analysis.

## 3. Results

### 3.1. µTBS

The mean and standard deviations of µTBS values are shown in Table 2. The result of a one-way ANOVA revealed that resin cements had a significant effect on the immediate µTBS (F = 22.59, *p* < 0.05) and after water storage µTBS (F = 22.83, *p* < 0.05). PV showed a statistically significant difference between HL and AP at 24 h after water storage. After 7 days of water storage, statistical differences were observed between all groups.

### 3.2. SEM Observation of the Failure Modes

**Figure 2:**
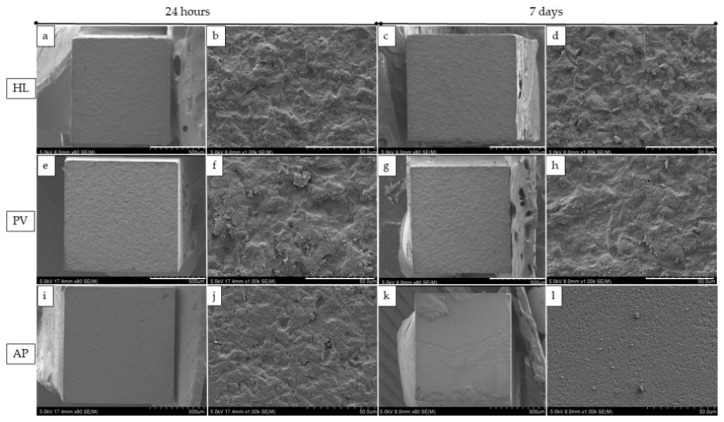
SEM images show the fractured dentin surface following the μTBS test (×80 and ×1000). HL, PV and AP represents the cohesive failure after 24 hours and 7 days. (**b**,**d**,**f**,**h**,**j**,**l**) represents higher magnification of (**a**,**c**,**e**,**g**,**i**,**k**) respectively.

The number of fracture modes after µTBS is shown in Table 2. For the HL group, the predominant failure mode at 24 h and 7 days after water storage was cohesive failure in cement at 93% and 87%, respectively. For the PV group, the predominant failure mode at 24 h and 7 days after water storage was cohesive failure in cement at 100% and 93%, respectively. For the AP groups, the predominant failure mode at 24 h and 7 days after water storage was cohesive failure in cement at 53% and 66%, respectively. Nevertheless, after 24 h, 20% of the beams showed a combination of interfacial failure in dentin and cement. Interfacial failure in dentin increased 27% after 7 days. Figure 2 shows the SEM images of the representative fracture modes at the dentin side at ×80 and 1000 magnifications. No pretest failure was observed in this study.

### 3.3. SEM Observation of Fillers and Elemental Analysis of Cured Resin Cements and Composite 

**Figure 3:**
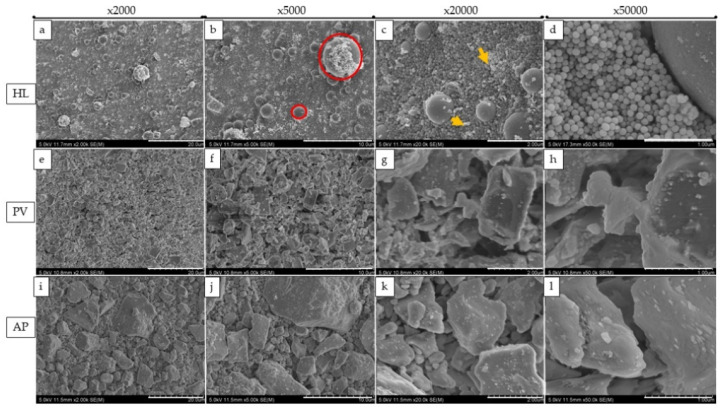
Representation of SEM images of the resin cement fillers. The rows from left to right present images with magnifications of ×2000, ×5000, ×20,000, and ×50,000, respectively; (**a**–**d**) represent HL; (**e**–**h**) represent PV; and (**i**–**l**) represent AP. The red circle represents a cluster with a regular distribution, and the yellow arrow represents nanometer-sized particles with a regular form.

**Figure 4:**
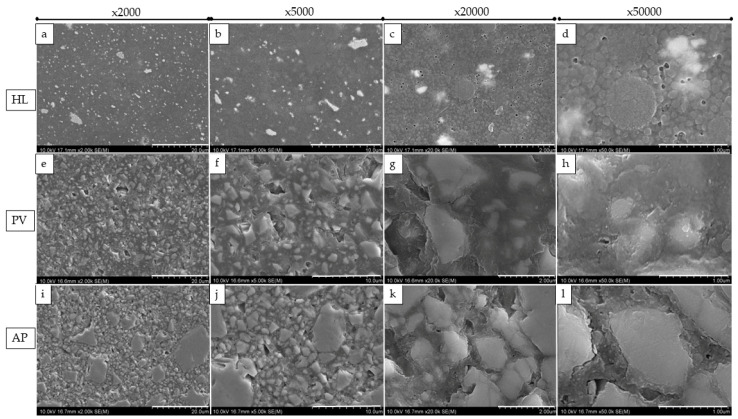
Representation of SEM images of resin cement fillers with polished surfaces. In order from left to right, each row displays a picture with a magnification range at ×2000, ×5000, ×20,000, and ×50,000. HL is indicated by (**a**–**d**); PV is represented by (**e**–**h**); and AP is indicated by (**i**–**l**).

SEM images at magnifications of ×2000, ×5000, ×20,000, and ×50,000 revealed changes in the size, shape, and distribution of various filler particles in the resin materials (Figure 3 and Figure 4). HL showed a combination of the regular-shaped filler (Figure 3d) and spherical-shaped filler (the diameter was approximately 100 nm) within the matrix (Figure 3a–c). The fillers were clustered together in some areas. On the other hand, PV and AP represent micro-hybrid filler particles with sizes that were approximately ranging from 0.50 μm to 6.30 μm, or 2.00 μm to 18.50 μm, respectively (Figure 3e,i).

**Figure 5:**
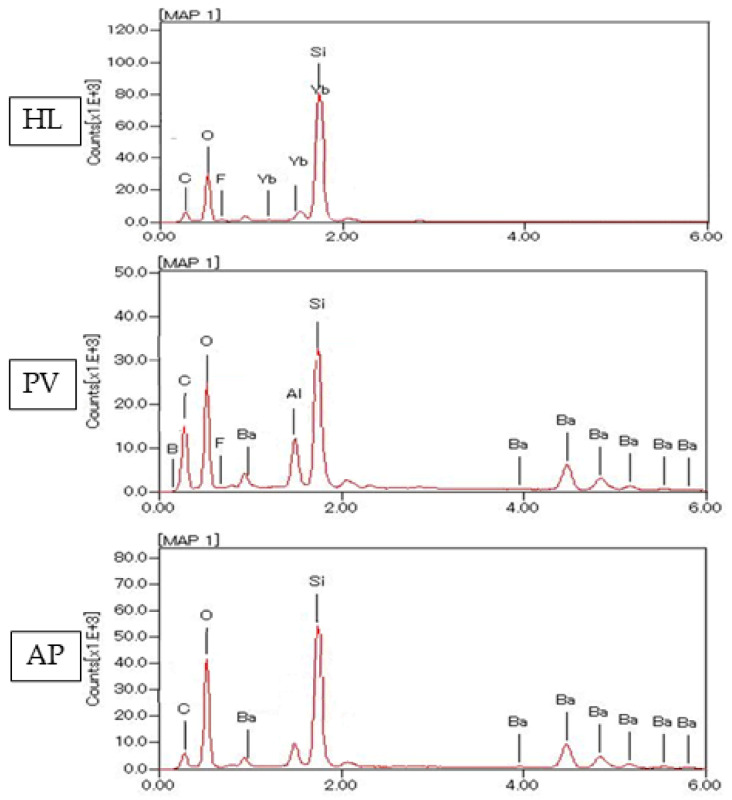
Elements identified by EDX spectroscopy microanalysis for novel experimental HL resin cement, PV resin cement, and AP resin composite.

**Figure 6:**
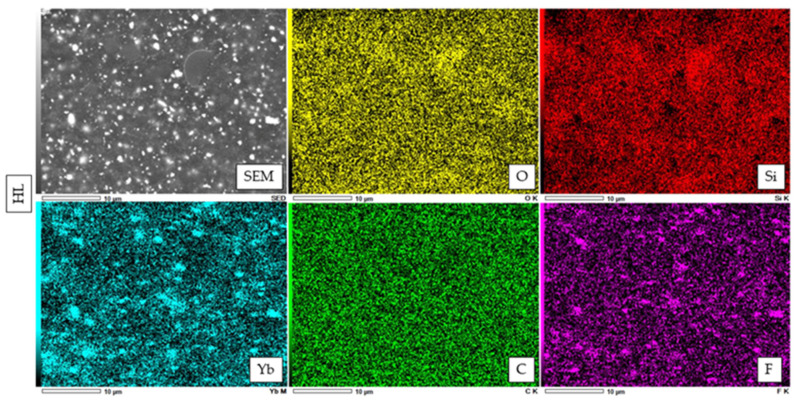
The representative EDX analysis picture of HL with a magnification of ×3000. The electron image obtained from the EDX analysis of HL reveals a widespread distribution of five significant elements. These elements are oxygen (O), silicon (Si), ytterbium (Yb), carbon (C), and fluorine (F).

**Figure 7:**
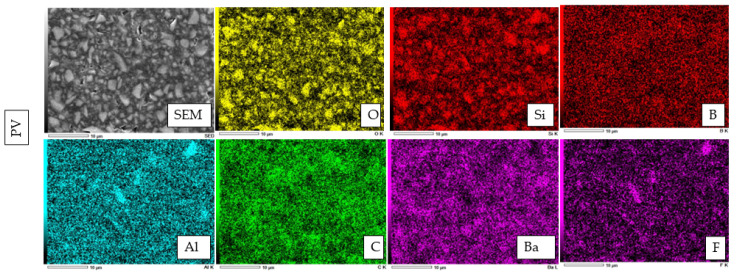
The representative EDX evaluation image of PV at ×3000 magnification. The electron image for EDX analysis of PL shows a wide distribution of seven important elements, including oxygen (O), silicon (Si), boron (B), aluminum (Al), carbon (C), barium (Ba), and fluorine (F).

**Figure 8:**
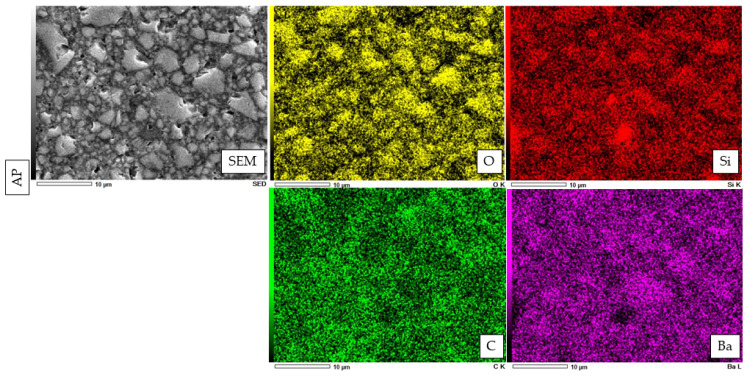
The representative EDX evaluation image of AP at ×3000 magnification.

An EDX element mapping evaluation and point analysis are shown in Figure 5, Figure 6, Figure 7 and Figure 8. From the results, F, Si, and Yb were detected in HL. Ba, C, O, and Si were detected in both PV and AP. Al, B, and F were detected only in PV.

The electron image for EDX analysis of AP shows the wide distribution of four essential elements, namely, oxygen (O), silicon (Si), carbon, and barium (B).

### 3.4. Microhardness Evaluation of Resin Materials

The means and standard deviation of KHN are shown in Table 3. A one-way ANOVA showed a significant difference in the KHN between the tested materials (*p* < 0.05) at 24 h and after 7 days of water storage. It is noteworthy that all the tested materials featured a different Knoop hardness. HL indicated the highest KHN when compared with PV. Then, AP showed the highest KHN among all tested materials.

## 4. Discussion

Light-cured resin cements are used with thin, translucent, indirect restorations permitting adequate light transmission [21]. The advantages of using light-cured resin cements include adequate working time, being able to remove excess cement without difficulties prior to polymerization, having the capacity to undergo “polymerization on demand”, and having greater color stability following cementation [22]. Recently, Kuraray Noritake dental corporation launched a new light-cured resin cement, named Panavia Veneer LC, which is unlike their already-existing, popular “Touch and Cure” resin cement, Panavia V5. According to the manufacturer, they incorporated newly developed spherical silica fillers, which provide better stability. They also incorporated the nano-cluster filler technology, which provides non-stickiness to the application tip and ensures easy application [23].

In the present study, HL demonstrated a significantly higher bond strength than PV, whereas AP exhibited a significantly higher bond strength than HL and PV. Therefore, the null hypothesis in terms of µTBS was rejected. In the present study, µTBSs were evaluated after 24 h and 7 days of water storage. For resin materials, the polymerization continues for at least 24 h after initiation. On the other hand, the µTBS was higher after 7 days, which was not surprising. This phenomenon might occur due to further polymerization after 24 h. Notably, AP was bonded with the dentin bonding agent SE. Recent studies reported that when AP is bonded with SE, it has high and stable bonding performance with dentin substrates [24,25]. The bonding agent and primer SE includes the functional monomer 10-methacryloyloxydecyl dihydrogen phosphate (10-MDP). The 10-MDP monomer is essential to bind with hydroxyapatite through chemical bonding, smear layer dissolution, and improved monomer penetration [26]. The higher µTBS of AP might be due to the presence of a photo-initiator system, which may have the potential to improve its degree of conversion and mechanical properties [25]. According to Politano et al., a light-cured resin composite also shows better mechanical properties and wear resistance than conventional dual-cured resin cement [27].

On the basis of our result, the commercially available “Touch and Cure” resin cement PV showed a significantly lower µTBS. One of the possible reasons for a lower µTBS is the slow polymerization of resin cement, which occurs because of the possible formation of water droplets in the adhesive cement interface when it comes into contact with dentin treated with an adhesive. It is possible that limited light transmission may allow water from the dentin to diffuse across the adhesive into the resin cement [28,29,30,31]. On the other hand, in contrast to PV, HL showed better bonding performance in our study. This might have occurred due to the improvement of the translucent structure of light-cured resin cement, thus allowing the resin cement to achieve better polymerization kinetics, which also contributes to the higher bond strength [32].

From our results, PV showed a significantly lower KHN than HL and AP. Therefore, the null hypothesis in terms of KHN was rejected. This might be because of the different material composition, filler size and morphology, and the distribution pattern or quality of the polymerization reaction [33]. 

From the EDX evaluation, Si indicated a silanated spherical silica filler in HL, which might allow better light transmission [34]. The observation of spherical-shaped filler particles might have a potential lubricating effect on the substance, which would allow the materials to flow more freely while having no effect on its viscosity [35]. It was demonstrated in a previous study that adding a silica filler to the resin materials might influence the radiopacity [36], and the addition of filler particles might have an influence on reduced degradation [37]. On the other hand, the SEM images show smaller filler particles in HL than PV and AP. This might have an impact on improving film thickness and allowing better bonding performance [38]. According to previous studies, the addition of Si fillers to resin materials resulted in higher bond strength, which is in accordance with this study [39,40,41].

The present study has some limitations. Although the outcomes of our investigation are promising, they ought to be regarded with caution for the time being. Furthermore, long-term in vitro bonding performance evaluation, water sorption and water solubility characterizations, color stability evaluation, and the degree of conversion are needed to verify the current results. 

## 5. Conclusions

Within the limitations of this in vitro study, it was concluded that the novel experimental light-cured resin cement HL showed better bonding performance than the conventional dual-cured resin cement PV, which might be due to its improved chemical structure. Moreover, innovative spherical-shaped silica fillers and smaller-sized filler particles might also contribute to the better bonding performance of the novel experimental light-cured resin cement.

## Figures and Tables

**Table 1:** Composition of the tested materials.

Materials	Compositions	Manufacturer	Lot No.
HL-100C	Paste: silanated spherical silica, UDMA, Ytterbium trifluoride, TEGDMA, hydrophilic aliphatic dimethacrylate, hydrophilic amide monomer, accelerators, dl-camphorquinone, pigments	Kuraray Noritake Dental, Tokyo, Japan	T200615-1
Panavia V5	Paste-A: Bis-GMA, TEGDMA, hydrophobic aromatic dimethacrylate, hydrophilic aliphatic dimethacrylate, initiators, accelerators, silanated barium glass filler, silanated fluoroalminosilicate glass filler, colloidal silica	Kuraray Noritake Dental, Tokyo, Japan	8H0168
	Paste-B: Bis-GMA, hydrophobic aromatic dimethacrylate, hydrophilic aliphatic dimethacrylate, silanated barium glass filler, silanated aluminum oxide filler, accelerators, dl-camphorquinone, pigments		
Clearfil AP-X	Paste: Bis-GMA, TEGDMA, silanated barium glass filler, silanated silica filler, silanated colloidal silica, dl-camphorquinone, catalysts, accelerators, pigments	Kuraray Noritake Dental, Tokyo, Japan	850124
Katana Avencia Block 2	Mixed filler with colloidal silica and aluminum oxide, cured resins consisting of methacrylate monomer (copolymer of UDMA and other methacrylate monomers), pigments	Kuraray Noritake, Tokyo, Japan	001122
Clearfil Ceramic Primer Plus	Ceramic primer: 3-trimethoxysilylpropyl methacrylate, MDP, ethanol	Kuraray Noritake Dental, Tokyo, Japan	2R0053
Panavia V5 Tooth Primer	Tooth primer: MDP, HEMA, hydrophilic aliphatic dimethacrylate, accelerators, water	Kuraray Noritake Dental, Tokyo, Japan	AW0071
Clearfil SE Bond 2	Primer: MDP, HEMA, hydrophilic aliphatic dimethacrylate, dl-camphorquinone, hydrophobic aliphatic, water	Kuraray Noritake Dental, Tokyo, Japan	4A0114
	Bond: MDP, Bis-GMA, HEMA, dl-camphorquinone, hydrophobic aliphatic dimethacrylate, initiators, accelerators, silanated colloidal silica		4H0173

Abbreviations: Bis-GMA—bisphenol-A-diglycidylmethacrylate; HEMA—2-hydroxyethyl methacrylate; MDP—10-methacryloxydecyl dihydrogen phosphate; TEGDMA—triethyleneglycol dimethacrylate; UDMA—urethane dimethacrylate.

**Table 2:** Mean ± SD of μTBS and fracture modes of resin cement.

Resin Cements	Mean ± SD (MPa) (24 h) (n = 15)	Fracture Mode (n) (24 h) IB/IB + C/IB + C + ID/CC/ID + C/ID	Mean ± SD (MPa) (7 Days)	Fracture Mode (n) (7 Days) IB/IB + C/IB + C + ID/CC/ID + C/ID
HL	60.32 ± 11.28 ^a^	0/0/0/14/1/0	53.81 ± 9.56 ^B^	0/0/0/13/0/2
PV	37.24 ± 7.28 ^b^	0/0/0/15/0/0	40.23 ± 8.78 ^C^	0/0/0/14/0/1
AP	72.07 ± 21.10 ^a^	0/0/0/8/4/3	67.24 ± 13.82 ^A^	0/0/0/10/1/4

Different superscript letters indicate statistically significant differences (Games–Howell test, *p* < 0.05). IB—interfacial failure at block; C—cement; CC—cohesive failure in cement; ID—interfacial failure at dentin.

**Table 3:** Means ± SD of KHN of the resin cements.

Cements	KHN (24 h)	KHN (7 Days)
HL	24.85 ± 3.39 ^a^	27.10 ± 4.90 ^a^
PV	11.01 ± 1.55 ^b^	10.87 ± 1.21 ^b^
AP	83.80 ± 14.43 ^c^	89.77 ± 14.08 ^c^

Different superscript letters indicate statistically significant differences (Games–Howell test, *p* < 0.05).

## Data Availability

The data presented in this study are available on request from the corresponding author.
